# Potential Therapeutic Effects of Natural Plant Compounds in Kidney Disease

**DOI:** 10.3390/molecules26206096

**Published:** 2021-10-09

**Authors:** Lorena Avila-Carrasco, Elda Araceli García-Mayorga, Daisy L. Díaz-Avila, Idalia Garza-Veloz, Margarita L Martinez-Fierro, Guadalupe T González-Mateo

**Affiliations:** 1 Molecular Medicine Laboratory, Academic Unit of Human Medicine and Health Sciences, Autonomous University of Zacatecas, Carretera Zacatecas-Guadalajara Km.6, Ejido la Escondida, Zacatecas 98160, Mexico; idaliagv@uaz.edu.mx (I.G.-V.); margaritamf@uaz.edu.mx (M.L.M.-F.); 2 Academic Unit of Human Medicine and Health Sciences, Therapeutic and Pharmacology Department, Autonomous University of Zacatecas, Zacatecas 98160, Mexico; emayorga3@gmail.com (E.A.G.-M.); daisydz27@gmail.com (D.L.D.-A.); 3 Research Institute of La Paz (IdiPAZ), University Hospital La Paz, 28046 Madrid, Spain; gtirma@gmail.com; 4 Molecular Biology Research, Centre Severo Ochoa, Spanish Council for Scientific Research (CSIC), 28049 Madrid, Spain

**Keywords:** natural plant compounds, kidney disease, renoprotective effects, bioactive compounds, clinical studies

## Abstract

Background: The blockade of the progression or onset of pathological events is essential for the homeostasis of an organism. Some common pathological mechanisms involving a wide range of diseases are the uncontrolled inflammatory reactions that promote fibrosis, oxidative reactions, and other alterations. Natural plant compounds (NPCs) are bioactive elements obtained from natural sources that can regulate physiological processes. Inflammation is recognized as an important factor in the development and evolution of chronic renal damage. Consequently, any compound able to modulate inflammation or inflammation-related processes can be thought of as a renal protective agent and/or a potential treatment tool for controlling renal damage. The objective of this research was to review the beneficial effects of bioactive natural compounds on kidney damage to reveal their efficacy as demonstrated in clinical studies. Methods: This systematic review is based on relevant studies focused on the impact of NPCs with therapeutic potential for kidney disease treatment in humans. Results: Clinical studies have evaluated NPCs as a different way to treat or prevent renal damage and appear to show some benefits in improving OS, inflammation, and antioxidant capacity, therefore making them promising therapeutic tools to reduce or prevent the onset and progression of KD pathogenesis. Conclusions: This review shows the promising clinical properties of NPC in KD therapy. However, more robust clinical trials are needed to establish their safety and therapeutic effects in the area of renal damage.

## 1. Introduction

**Figure 1:**
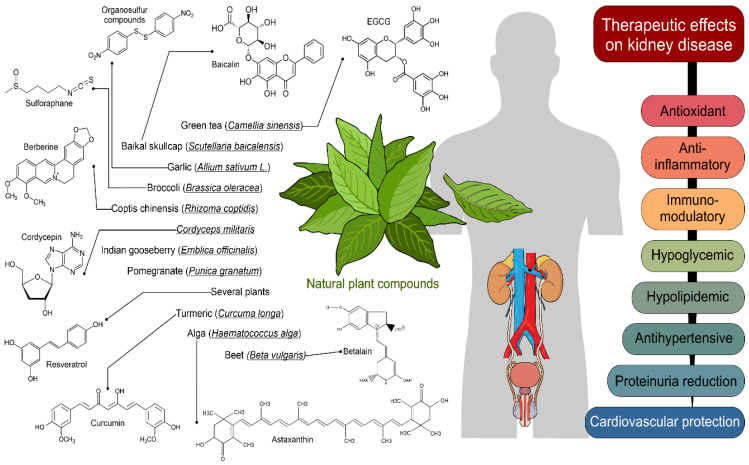
Therapeutic effects of natural plant compounds on kidney disease. Some elements of this figure were taken from the Mind the Graph platform, available at www.mindthegraph.com of access; 30 September 2021).

Historically, natural plant compounds (NPCs) have been used as treatments and their chemical structures could give rise to new therapeutic options [1]. NPCs are active substances isolated from plants that can modulate several pathways, including the epithelial-to-mesenchymal transition (EMT) via antioxidant, anti-inflammatory or anti-fibrotic mechanisms [1]. Chronic kidney disease (CKD) is a recurrent public health problem worldwide [2]. Several diseases (e.g., hypertension, diabetes, cancers, infection, drug-induced nephrotoxicity) may disturb kidney function, eventually leading to CKD development. Around the world, the prevalence of CKD is 13.4% (11.7–15.1%) and it has been estimated that patients with end-stage renal disease (ESRD), which requires kidney substitution therapy, number between 4.902 and 7.083 million, making it a leading cause of death around the world [2,3]. The higher number of CKD patients over recent years has spurred great interest in searching for efficient approaches to avoid or decrease the development of CKD and its progression to ESRD. Traditional treatments provide limited kidney protection, so new therapeutic compounds are necessary. Currently, inflammation is recognized as a key unfavorable pathway in the progression and development of CKD; consequently, new anti-inflammatory compounds targeting or directed towards specific molecular signatures may be promising therapeutic approaches for CKD. NPCs derived from herbs or medicinal plants have become important in preclinical and clinical research for the development of such targets. Several NPCs have been shown to have a renoprotective effect and improve the outcomes of disorders present in various categories of CKD in clinical studies, mainly through activating antioxidant defense systems and decreasing proinflammatory signaling pathways (Table 1; Figure 1) [3]. This review collected comprehensive evidence of the kidney-protective potential of bioactive plant compounds.

## 2. Methods

This systematic review is based on pertinent papers revealed by a selective search using relevant keywords that were collected from databases such as Science Direct, EBSCO, Scopus, and PubMed to analyze clinical trials focused on the potential impact of natural bioactive compounds regarding their therapeutic efficacy as a treatment for renal diseases. The principal objective of this review of the effects of bioactive natural compounds on kidney damage was to reveal the efficacy demonstrated in clinical studies.

## 3. Results

### 3.1. Molecular Mechanisms Involved in Kidney Damage

**Figure 2:**
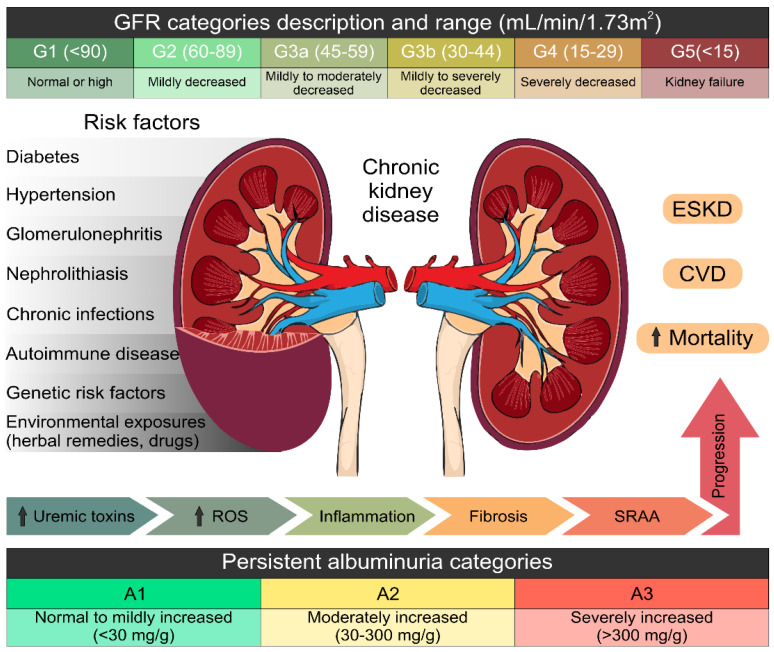
Causes, definition and prognosis of chronic kidney disease classed by glomerular filtration rate and albuminuria. Categories are grouped by risk of progression, defined by a decline in GFR category. Adapted from the Kidney Disease: Improving Global Outcomes (KDIGO 2012) categories of the National Kidney Foundation (NFK). ESKD, end-stage kidney disease; CVD, cardiovascular disease; CKD, chronic kidney disease; GFR, glomerular filtration rate. Some elements of this figure were taken from the Mind the Graph platform, available at www.mindthegraph.com. (Last date of access; 30 September 2021).

Kidney damage is a global public health problem that has been growing worldwide. Renal diseases involve acute kidney injury (AKI) and CKD, which can lead to ESRD. Consequently, patients needing renal replacement therapy are estimated to number between 4.902 and 7.083 million [2,3]. The worldwide projected prevalence of CKD is 13.4% (11.7–15.1%) [2]; likewise, in 2017, the global prevalence of CKD was 9.1%, i.e., approximately 700 million cases [29]. CKD is frequently asymptomatic with fast progression and is commonly associated with hypertension [30] and Type 2 diabetes mellitus (T2DM). These diseases are considered the leading causes of the development of CKD and ESRD [31]. Nevertheless, CKD is a chronic disorder characterized by albuminuria (>30 mg) for 24 h, a reduced glomerular filtration rate (GFR) (<60 mL/min/1.73 m^2^) for more than 3 months, and progressive glomerular, tubular, and interstitial damage [32,33,34]. The prognosis of CKD is classified according to the GFR and albuminuria categories, GF grades (G1 to G5) and albuminuria grades (A1 to A3) (Figure 2), ccording to the Kidney Disease: Improving Global Outcomes guidelines (KDIGO 2012), [35]. 

**Figure 3:**
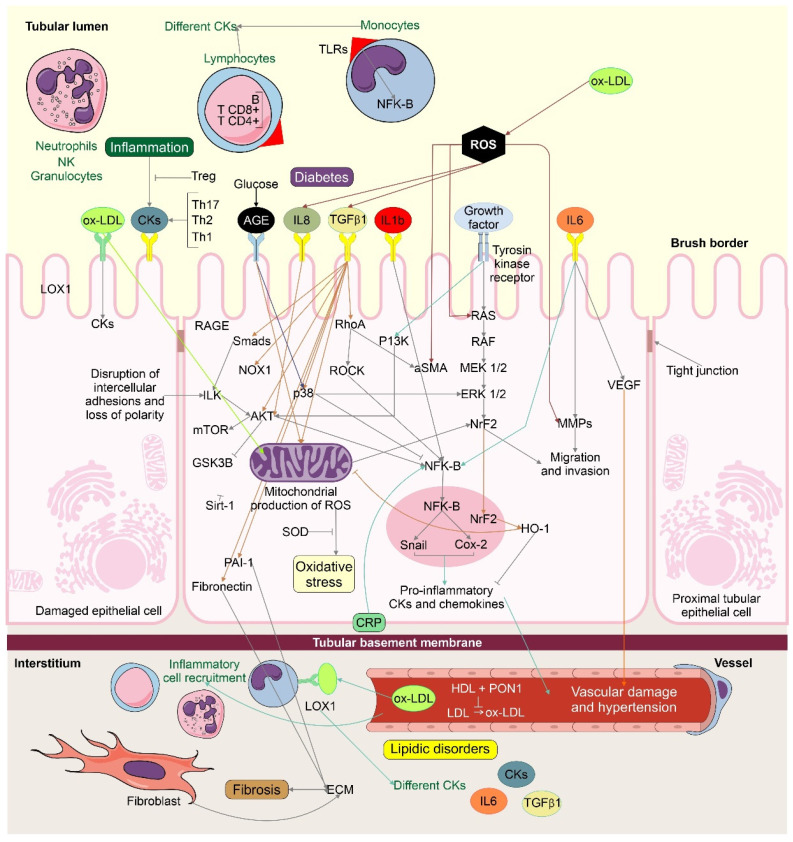
Alterations related to kidney injury, including diabetes, hypertension, and vascular damage (CVD and CAD) and the molecular mechanisms involved in inflammation, fibrosis, lipid disorders, and oxidative stress (highlighted in boxes). Some elements of this figure were taken from the Mind the Graph platform, available at www.mindthegraph.com (Last date of access; 30 September 2021).

Numerous molecular signaling pathways are implicated in the onset and progression of renal damage, including the renin–angiotensin system (RAS), which is the central regulator of kidney fibrosis. Together, tissue and circulatory RAS are believed to be overstimulated in the progress of kidney fibrosis stimulation. RAS increases levels of angiotensin II (Ang II), which play a dominant role in kidney fibrosis by mediating the liberation of transforming growth factor-β (TGF-β) and starting inflammatory processes [36,37,38,39]. Likewise, Ang II and inflammation may be the key factors in the glomerulotubular pathogenic response and have been related to microalbuminuria and development of kidney damage [40]. In patients with diabetes and severe hyperglycemia, many induced metabolic and hemodynamic disorders occur, including the increased development of advanced glycation end products (AGEs), augmented reactive oxygen species (ROS) generation, and initiation of the protein kinase C (PKC) and polyol pathway, which are believed to induce the development and evolution of diabetic nephropathy [39]. Anther molecular pathway contributing to kidney damage is the formation of AGEs, which promote intracellular nitric oxide (NO) and ROS, and the mitogen-activated protein kinase (MAPK) cascade. The creation of AGEs could induce kidney damage by altering the role of proteins, oxidative stress (OS), pro-inflammatory cytokines, and growth factors [41,42,43]. In addition, the AGE–RAGE interaction induces activation of the nuclear factor kappa-light-chain-enhancer of triggered B cells (NF-kB) and activation of MEK and MAP kinases, increasing intracellular OS by stimulating NADPH-oxidase, an important regulator in superoxide radical generation [44,45]. Likewise, ROS and free radicals may respond to membrane lipids, nucleic acids, and proteins, and start to produce cellular injury. When ROS are created in excess quantities, OS, antioxidant defense mechanisms, and cell damage can occur, which accelerates the progression of CKD. Other pathways associated with CKD pathogenesis are stress stimuli and the activation of profibrotic growth factors such as TGF-β1, connective tissue growth factor (CTGF), and hydrogen peroxide (H_2_O_2_). In addition, TGF-β has been demonstrated to increase ROS production and to decrease the antioxidant system, thus inducing OS and/or redox imbalance. Essentially, redox imbalance significantly helps TGF-β’s pathophysiologic properties such as fibrosis [46,47]. TGF-β may drive towards kidney fibrosis through accumulation of the extracellular matrix (ECM) and activation of epithelial dysfunction and pro-inflammatory reactions. TGF-β is the most powerful stimulator of the EMT, and this can induce this process in the epithelial cells of various organs in vivo and in vitro [48,49,50,51]. Signaling pathways such as the MAPK, Smad, and PI3K pathways are related to the stimulation of EMT by TGF-β [52,53]. Essentially, ROS come from many sources, such as the mitochondria or NOXs, which have been exposed to TGF-β-induced EMT at the beginning of fibrosis and cancer [49,50,54,55,56]. Recent evidence has shown the process of linking ROS to TGF-β-activated EMT in the context of fibrosis [48,49,57]. Rhyu et al. discovered that NADPH oxidase-originated ROS are upstream signaling particles in TGF-β1-stimulated fibronectin and production of the plasminogen activator inhibitor-1 (PAI-1). This step happens throughout MAPK stimulation in the tubular epithelial cells [57]. Likewise, other research has suggested that ROS play a significant role in TGF-β1-activated EMT, mainly through MAPK stimulation [58,59]. These pathways overlap and interact with each another, thereby altering their biological activities, which promotes the evolution of kidney fibrosis and exacerbates kidney damage (Figure 3). Consequently, any pharmacologic agents that are capable of preventing these disadvantageous pathways can be thought of as renoprotective and a potential therapeutic approach in the control of kidney damage.

### 3.2. Potential Renoprotective Effects of Some Natural Plant Compounds

#### 3.2.1. Allicin (Diallyl Thiosulfinate)

The main bioactive constituents of garlic (*Allium sativum* L.) are organosulfur compounds (OSCs). Among these compounds, the most plentiful sulfur composite contained in fresh and dry garlic is alliin (S-allyl-l-cysteine sulfoxide) [60,61]. Alliin can rapidly change into allicin (diallyl thiosulfinate) [61], which has been described as a major OSC and has been reported to be one of the primary substances responsible for antiviral activity (33), immunomodulatory, anti-inflammatory [62], antioxidant [63], and other pharmacological properties [61]. Alliin also contains non-sulfur constituents that probably have synergistic or additive properties with OSCs [64].

A randomized double-blind clinical trial study evaluated the effect of garlic extract on the serum inflammatory markers of 42 subjects undergoing peritoneal dialysis (PD), a renal failure therapy. The patients received a dose of 400 mg of a standardized garlic extract, which was made in the shape of tablets including 1 mg (1000 mcg) of alliin. The dosing scheme in the cases group (garlic extract group) was twice a day for 8 weeks, while the control group received standard treatments plus a placebo during the same period. The results showed that in the patients who received the garlic extract, the inflammatory markers IL-6, C reactive protein (CRP), and the erythrocyte sedimentation rate (ESR) were all significantly reduced, while in the placebo group, a significant decrease was only observed in IL-6. However, the assessment of these effects in larger trials is strongly recommended [4].

#### 3.2.2. Astaxanthin 

Xanthophyll carotenoid colorant that has beneficial properties, including anticancer, antioxidant, and anti-inflammatory properties. The most common source of astaxanthin (AST; 3,3′-dihydroxy-β,β’-carotene-4,4′-dione) as used in nutritional supplements is obtained from *Haematococcus alga* [65]. The Food and Drug Administration (FDA) of the United States of America has accepted astaxanthin as a nutraceutical [66]. Studies in humans have discovered notable decreases in OS, dyslipidemia, and inflammatory markers after oral administration of astaxanthin. A clinical evaluation of gastric inflammatory biomarkers was conducted in subjects with functional dyspepsia treated with astaxanthin and revealed a significant increase in CD4+ cells and a decrease in CD8+T cells in 21 patients with *Helicobacter pylori* (*H. pylori*) treated with 40 mg of astaxanthin daily and 23 patients were administered a placebo, so the authors suggested that these differences indicated a greater change to a humoral immune response instead of a cytotoxic response [67]. Moreover, they explained that in animal models, where the diet can be standardized without antioxidants, astaxanthin had a tremendous effect on inflammation and on the density of *H. pylori* [68].

Likewise, a randomized placebo-controlled trial was conducted to investigate the possible action of astaxanthin administration on lipid peroxidation, adiponectin levels, glycemic control, anthropometric indices, and insulin sensitivity in subjects with T2DM, which is a common cause and driver of kidney damage. In the study group, after 8 weeks of 8 mg of astaxanthin administration, the serum adiponectin concentration increased and there was a reduction in visceral body fat mass (*p* < 0.01), serum triglycerides, very-low-density lipoprotein cholesterol (VLDLc) levels, and systolic blood pressure [5]. The investigators suggested that astaxanthin may provide vascular benefits and reduce the indicators of OS and inflammation [69]; however, their results showed that 12 mg oral astaxanthin/day for 12 months had no great effect on arterial stiffness, OS, or inflammation in renal transplant recipients [70]. Nevertheless, it has been proven in animal models that astaxanthin has a protective effect on kidney damage by regulating inflammation (inducing CD8+ T cells) [71] and oxidative stress-related NRF2/KEAP1 and ROS pathways [72]. Thus, although astaxanthin has demonstrated antioxidant, anti-inflammatory, and vascular-protective effects on different pathologies, more studies would be interesting to elucidate its capacity to improve CKD pathogenesis in human beings.

#### 3.2.3. Baicalin 

It is a flavone glycoside isolated from the roots of *Scutellaria baicalensis*. Clinical studies with baicalin as an adjunctive treatment have targeted the protective functions in liver fibrosis, ulcerative colitis, and diabetes mellitus [73,74]. Many studies have researched the potential of baicalin in the management of subjects with early diabetic renal injury [75]. The effect of baicalin was analyzed in subjects with diabetic nephropathy at a dose of 800 mg three times per day, whereas the control group received a placebo. Both groups were treated and studied for 6 months. The results indicated that baicalin had the potential to decrease the level of proteinuria and improve the kidney function of the diabetic subjects, since after taking baicalin therapy, the superoxide dismutase (SOD) and glutathione peroxidase (GSH-px) concentrations of patients were clearly elevated, and the aldose reductase (AR) activity, NF-κB, and vascular endothelial growth factor (VEGF) content reduced considerably. These results demonstrated that baicalin at a dose of 800 mg three times a day can reduce renal vascular permeability, enhance the kidney function of subjects with diabetic nephropathy, and delay the progress of diabetic nephropathy via the polyol pathway, and the antioxidative stress, anti-inflammatory, and other pathways. Further studies are needed to evaluate the nephroprotective effects of baicalin through other mechanisms [8].

A clinical study aimed to evaluate the actions of baicalin on AKI in pediatric sepsis. This clinical investigation included 50 pediatric patients with a diagnosis of sepsis, 25 of whom were given an adjunctive treatment of oral baicalin for 15 days and the other 25 patients received only standard therapies. Their results showed that neither the blood urea nitrogen (BUN) nor the serum creatinine concentrations of the control group changed significantly after basic therapy, but they decreased considerably in the baicalin adjunctive therapy group. It can be assumed, based on these two indices, that the renal function of the baicalin group improved, probably due to the baicalin adjunctive therapy. Therefore, baicalin may decrease AKI in pediatric patients with sepsis. Although this study demonstrated the protective effect of baicalin against AKI in this study group, shown as a decrease in BUN and creatinine levels, the authors did not report the dose administered and therefore the appropriate dosage and probable complications need to be evaluated in subsequent studies [76].

#### 3.2.4. Betalain

Another NPC with interesting properties in renal disease is betalain. Betalains are divided into two groups: betacyanins, which produce red tones and are formed by condensation of a cyclo-DOPA (dihydroxyphenylalanine) structure with betalamic acid, and betaxanthines, which produce yellow coloration and are synthesized from different amino compounds and betalamic acid [77]. A pilot crossover clinical trial evaluated whether dietary administration of a betalain-rich extract from red beetroot and a betacyanin-rich extract of *Opuntia stricta* fruits had the ability to modulate genes/protein expression in coronary artery disease (CAD) patients. CKD is a major risk factor for CAD. Patients with CKD display a high prevalence of hypertension, and cardiovascular disease (CVD) is the leading cause of morbidity and mortality in these patients. The high prevalence of traditional CAD risk factors, such as diabetes and hypertension, mean that such patients are also exposed to other non-traditional uremia-related CVD risk factors, including inflammation, oxidative stress, and abnormal calcium–phosphorus metabolism [78].

In a pilot randomized crossover trial, 48 male coronary artery disease patients received about 50 mg of betalain or betacyanin daily for 2 weeks on three occasions separated by washout periods. Patients were divided into three groups depending on the supplement: a betalain-rich supplement of red beetroot (*Beta vulgaris*), a betacyanin-rich supplement of prickly pear cactus (*Opuntia stricta*), and a placebo. The results showed that betalain increased sirtuin-1 (SIRT1) and reduced lectin-like oxidized LDL receptor 1 (LOX1) and highly sensitive C reactive protein (hs-CRP) in the peripheral blood mononuclear cells (PBMCs) of patients. These results may be due to the reduction in OS and inflammation via the antioxidant and anti-inflammatory properties of betalains. Thus, betalains may be promising alternatives for supplementing therapies in OS, inflammation, and aging-associated diseases. However, additional analyses are necessary to gain a deeper understanding of their specific physiological functions [9,79,80].

#### 3.2.5. Beetroot Juice

It’s a source of concentrated inorganic nitrates. One study on CKD patients (CKD Stage II–IV according to the K/DOQI (Kidney Outcomes Quality Initiative) guidelines) by Kemmner et al. suggested that the administration of beetroot juice with a nitrate load of 300 mg to nine patients increased nitric oxide (NO) concentrations and reduced the renal resistive index (RRI), which are prognostic markers for cardiovascular mortality, compared with a placebo [10]. This result was more evident among CKD patients with decreased renal function and increased arterial stiffness, who had GFR values below the normal range. This reduced value was mainly caused by diabetic or hypertensive renal impairment, both of which are factors responsible for the subsequent outcome of kidney failure. Compared with the controls, the serum creatinine, potassium, and GFR serum levels did not change considerably after beetroot juice ingestion. The serum potassium concentration/level was similar to the placebo group. The results established the improving action of *Beta vulgaris* as a helpful therapeutic option on the indicators of renal function, decreasing the gradual rate of kidney damage and subsequent mortality in high-risk groups involving hypertensive and diabetic patients with nephropathy [10].

#### 3.2.6. Berberine (BBR)

It is an isoquinoline alkaloid and is the main active compound isolated from *Rhizoma coptidis* and *Cortex phellodendri*. New analyses have demonstrated that berberine has numerous pharmacological benefits, such as lowering blood glucose, antioxidant activity, blood lipid regulation, reduced inflammation, and elevated insulin sensitivity, thus improving insulin resistance [81,82]. Recently, it has been described as a potential anti-diabetic nephropathy drug [83]. The urine microalbumin/creatinine ratio (UACR) and GFR are significant indicators used for evaluating the status of diabetic nephropathy. A randomized controlled clinical trial was conducted to look at the effects of berberine effects serum Cys C and UACR in patients with T2DM. The researchers administered berberine at a dose of 0.4 g three times per day for a period of 6 months. Their results showed that berberine improved diabetic kidney disease by reducing UACR and serum Cys C in T2DM patients, and the results were statistically significant. However, the authors mentioned that the number of cases in their study was small and the observation period was not long enough, so it is indispensable to verify the long-term efficacy and safety of berberine in the progression of CKD [11]. Berberine has been shown to protect renal tubular cells against hypoxia/reoxygenation injury via Sirt1 [84].

#### 3.2.7. Cordycepin

It is a naturally derived active compound produced by *Cordyceps militaris*, belonging to the *Clavicipitaceae* family. It is a fungus with a long history of common use in traditional medicine and its specific compound, cordycepin, has several health-promoting properties, including anticancer, anti-inflammatory, immunomodulatory, antidiabetic, and antiobesity effects [85,86,87]. Furthermore, antidiabetic and nephroprotective effects have been attributed to cordycepin in experimental studies [88]. In a clinical study with CKD patients, *Cordyceps militaris* was administered at 100 mg daily and compared with a placebo (control group). *Cordyceps militaris* decreased the protein concentration of TLR4, NF-κB p65, COX2, IL-1β, and TNF-α. Their results showed that eGFR was considerably improved compared with the control group after 3 months of treatment. This showed that *Cordyceps militaris* improved the eGFR of CKD patients, and the outcomes suggested that *Cordyceps militaris* ameliorated kidney function and controlled blood levels of urinal protein, BUN, and creatinine. This study provided support for the possibility that *Cordyceps militaris* controlled CKD evolution by controlling the TLR4/NF-κB redox signaling pathway [12]. 

#### 3.2.8. Curcumin 

It is a component of turmeric (*Curcuma longa*). Curcumin (diferuloylmethane) has been shown to be a TNF blocker in vitro and in vivo; nevertheless, only a limited number of analyses have confirmed that curcumin is efficient at reducing TGF- β, IL-8, and TNF-α levels in preclinical [89] and clinical studies [90]. There are several causes involved in the pathogenesis of diabetic renal damage, but TGF-β is considered to be a key player in the progress of actions towards ESRD. In a randomized double-blind placebo-controlled study, the effects of turmeric on TGF- β, IL-8, and TNF-α levels, as well as proteinuria in the urine and serum, were investigated in patients with T2DM nephropathy (*n* = 20) and a control group (*n* = 20). Individually, the test group subjects were given one capsule containing 500 mg turmeric, 22.1 mg of which was the active component curcumin, administered with each meal (three capsules daily) for 2 months. Their results demonstrated that serum TGF-β and IL-8 values were considerably lower post-turmeric administration, and that short-term turmeric supplementation can reduce proteinuria. Furthermore, they did not report any adverse effects associated with turmeric intake during the 2-month trial duration [17]. The increased ROS may induce IL-8 production and result in reduced glutathione levels, which may be due to increased OS caused by inflammation in ESRD patients with and without diabetes mellitus [91]. In addition, redox imbalance contributes importantly to TGF-β production [47], which has long been considered a key mediator of renal fibrosis. 

Another study suggested the possible efficacy and safety of turmeric in reducing uremic pruritus (UP) and hs-CRP in ESRD patients. Nevertheless, the authors described that a larger sample size and a longer therapy period would be required to additionally confirm the long-term efficacy and safety of adding turmeric in the hemodialysis (HD) population [18]. A review showed that curcumin, as an antioxidant, decreased renal inflammation and could prevent the deleterious complications of diabetes [92]. It has been shown that it is a secure auxiliary therapy for improving macroscopic proteinuria in T2DM patients [93].

Other studies have also shown that curcumin was an effective adjuvant therapy for decreasing macroscopic proteinuria, as was shown in a randomized double-blind clinical trial performed on 46 patients with T2DM. In this study, the patients received 500 mg (one capsule) of curcumin three times/day after meals for 16 weeks. [94]. The researchers mention that the range of persistent proteinuria was closely associated with the level of deterioration of creatinine clearance; curcumin reduced creatinine clearance, resulting in slower kidney function impairment and, possibly, a reversal of fibrotic injury [94]. Supplementation with 6 g of turmeric augmented postprandial serum insulin levels but did not seem to affect plasma glucose levels or the glycemic index in healthy persons. These results suggest that turmeric might stimulate insulin secretion [95]. On the other hand, in a pilot study, researchers demonstrated that curcumin reduced lipid peroxidation in the plasma of individuals with non-diabetic or diabetic proteinuric CKD and enhanced the antioxidant activity in subjects with diabetic proteinuric CKD, demonstrating that dietary administration of turmeric has a potential antioxidant effect in patients with non-diabetic or diabetic proteinuric CKD [15]. 

Additionally, a randomized placebo-controlled trial examined the effects of curcumin and quercetin on early graft function in 43 dialysis-dependent cadaveric renal recipients. A single capsule of curcumin (480 mg) and quercetin (20 mg) was administered to patients for 1 month after the transplant operation. The instigators of this report concluded that curcumin and quercetin may restore early outcomes in cadaveric kidney transplantation, possibly through activation of heme oxygenase-1 (HO-1) [19]. A likely use of the advantageous properties of these bioflavonoids is the activation of HO-1, an inducible enzyme that produces carbon monoxide. Stimulation of HO-1 in organ transplantation has the capacity to decrease ischemia-reperfusion (IR) damage and alloimmunity. Furthermore, curcumin induced HO-1 mRNA in human proximal tubule renal cells [96], and this induction may be dependent on the nuclear factor erythroid 2-related factor 2 (Nrf2) transcription factor [97]. 

In the same way, short-term turmeric administration may decrease hematuria, proteinuria, and systolic blood pressure in subjects with relapsed or refractory lupus nephritis, as established in a randomized and placebo-controlled trial of 24 patients with this biopsy-proven pathology. Each patient in the test group was given one capsule for 3 months, providing 500 mg turmeric, 22.1 mg of which was the active element curcumin (three capsules daily). Nevertheless, long-term tests with higher doses of turmeric are required to elucidate its outcomes on the renal function of such patients and the rate of development of CKD of different origins [17]. However, further research is recommended to evaluate the short- and long-term safety and efficacy of curcumin in this study group [98].

#### 3.2.9. Epicatechin-3-gallate, Epicatechin, Epigallocatechin 

Polyphenolic constituents (from tea plant; *Camellia sinensis*) have high anti-inflammatory, antioxidant, and antimutagenic properties in various biological systems. Polyphenols display potential beneficial health properties for chronic diseases, including CKD [99]. The majority of ingredients in tea (among the 400 chemicals that have been identified) are polyphenolic compounds, especially flavonoids originating from the tea plant (*Camellia sinensis*), which is high in catechin (flavonoid subtype). Three major catechins found in green tea have been described: epicatechin, epigallocatechin, and epicatechin-3-gallate (EGCG). The most abundant and extensively investigated catechin is EGCG [99]. In recent times, the potential use of EGCG in the treatment and prevention of numerous renal diseases, which are often related to inflammation and oxidative stress, has been reviewed [100]. 

The antioxidant, anti-inflammatory, and antiapoptotic activities of EGCG hold strong hope for its use as an alternative approach to the management or prevention of several renal diseases. The beneficial effects of EGCG are mediated by the underlying molecular mechanisms, mainly by direct inhibition of stress or stimulus-induced ROS overproduction; in addition, it could impact the Nrf2-Keap1-Cul-3 complex, resulting in nuclear translocation of free Nrf2, subsequently binding to the antioxidant response element (ARE) inside the promoter region of the cytoprotective genes and those encoding antioxidant enzymes, which are likewise modulated by NF-κB signaling pathways. Nevertheless, the majority of all studies using EGCG or green tea in kidney diseases have been in animal models or cell cultures. Therefore, clinical studies are needed to obtain scientific support for the renoprotective properties of EGCG on renal pathologies [101,102].

In other research, the authors evaluated the possibility of using a mixture of EGCG and amla extract (AE) obtained from *Emblica officinalis*, an Indian gooseberry, in the treatment of uremic subjects with T2DM. An EGCG/AE tablet was administered orally (one tablet three times a day) to uremic diabetic patients for 3 months, using a total daily dose of 300 mg of EGCG and 300 mg of AE/day. The results showed that 1:1 EGCG/AE improved diabetic biomarkers, antioxidant protection, and the atherogenic index in uremic diabetic subjects. Based on these results, the researchers concluded that EGCG and AE has the potential for adjuvant use in the treatment of diabetic patients in a uremic state [21,103].

#### 3.2.10. Pomegranate (*Punica granatum*) 

A fruit designated as “a medicine in itself” that has long been included in traditional remedies for preventive and therapeutic purposes [83]. It has a high content of polyphenols, alkaloids, and anthocyanins (flavonoid antioxidants), which are highly effective at scavenging free radicals [104,105]. The nephroprotective effects of pomegranate extract on calcium-containing lithiasis development in patients aged 18 to 70 years with recurrent stone formation have been medically reviewed. Daily administration of pomegranate extract stimulated a significant increase in serum paraoxonase1 (PON1) activity, along with a decrease in calcium oxalate supersaturation. PON1 is an anti-atherosclerotic component associated with high-density lipoprotein (HDL). An important function of PON1 is to prevent the oxidation of both HDL and LDL [106]. Low levels of PON1 have been associated with hypercholesterolemia, diabetes, and vascular diseases. Supported by the aforementioned findings, the researchers suggest that this strategy could potentially control the risk of kidney stone development [23].

#### 3.2.11. Resveratrol

It is a phenolic substance (a non-flavonoid stilbene polyphenol), and the trans-isomer is considered to be the most biologically active form. Numerous preclinical and clinical analyses have recognized the anti-inflammatory, antidiabetic, hepatoprotective, neuroprotective, anticancer, and antioxidant properties of resveratrol (RSV; 3,5,4′–trihydroxystilbene). Likewise, supporting in vivo and in vitro tests of RSV in renal damage indicated that it can decrease fibrosis, mesangial expansion, OS, and inflammatory cytokine levels, while improving renal structure and function [107]. Furthermore, with the aim of evaluating the actions of RSV administration on Nrf2 and NF-κB expression in non-dialyzed patients with CKD, the investigators performed a randomized double-blind crossover trial in 20 non-dialyzed patients with CKD, and their results showed that RSV administration at a dose of 500 mg per day for a period of 4 weeks had no antioxidant or anti-inflammatory activity in those subjects [108]. On the other hand, in another randomized double-blind trial, peritoneal dialysis patients were administered a low (150 mg/day) or high (450 mg/day) dose of trans-resveratrol over 12 weeks, resulting in an intensification in the average net ultrafiltration (UF) volume and level. Moreover, angiogenesis biomarkers, VEGF, fetal liver kinase-1 (Flk-1), and the angiopoietin (Ang)-2 rate in peritoneal dialysate effluent (PDE) became markedly diminished in patients treated with a high dose of RSV, whereas the levels of angiopoietin receptor (Tie-2) and thrombospondin-1 (Tsp-1) in the effluent were augmented with RSV treatment. This information implied that RSV administration had angiogenesis-enhancing results in PD patients and augmented ultrafiltration renal function [26]. Moreover, RSV treatment significantly decreased serum creatinine concentrations and preserved GFR, suggesting improved renal function. Therefore, the researchers suggested that RSV lowered insulin resistance and OS and increased pAkt:Akt levels in platelets and urinary ortho-tyrosine elimination [25].

Likewise, an analysis was conducted of 24 patients diagnosed with hypertension between 45 and 65 years of age and with underlying endothelial damage who participated in a randomized double-blind placebo-controlled crossover trial. Each patient was administered a single dose of trans-resveratrol (300 mg) or a placebo. Measurements of blood pressure (BP), aortic systolic blood pressure (SBP), and brachial flow-mediated dilation (FMD) were monitored before and 1.5 h after the intervention. The key results reported were that FMD was considerably augmented in female but not male patients administered trans-resveratrol. These results suggest that hypertensive patients with endothelial dysfunction, mainly women and those with high LDL-c, showed an improvement in endothelial function with a single dose of trans-resveratrol, although there were no significant improvements in peripheral and central BP ranges [27,109]. 

Furthermore, in another randomized double-blind placebo-controlled clinical trial conducted in 60 patients with a diagnosis of T2DM and albuminuria, resveratrol was randomly administered at a dose of 500 mg per day or a placebo for a period of 90 days, and losartan was additionally supplemented at dose of 12.5 mg per day to all research subjects. Their results showed that the mean urine albumin/creatinine concentrations were significantly reduced in the RSV group, and urinary albumin clearance, fasting plasma glucose (FPG), insulin, homoeostasis model evaluation of insulin resistance (HOMA-IR), and glycosylated hemoglobin (HbA1c) all decreased considerably in the RSV group compared with the placebo group, while the antioxidant effect of RSV was estimated by measuring serum levels of SOD1, glutathione peroxidase (GSH-Px), and catalase (CAT). Patients treated with RSV showed significant increases in serum levels of SOD1, GSH-Px, CAT, and NO compared with the placebo, confirming its antioxidant actions. The authors concluded that RSV could be effective as an adjunct to angiotensin receptor blockers (ARBs) to diminish urinary albumin excretion in subjects with diabetic nephropathy [24].

As indicated, the health benefits of RSV seem to be extensive, with the reduced side effects of RSV making it an interesting option for therapeutic use targeting renal damage. However, further research and clinical trials are indispensable for completely comprehending the actions of RSV on renal injury [107,110].

#### 3.2.12. Sulforaphane 

It is a bioactive component that is a precursor of glucosinolate in cruciferous vegetables, particularly in young broccoli sprouts (BS) [111,112]. The probable actions of sulforaphane (SFN; 1-isothiocyanate-4-methylsulphinylbutane) in CKD involve the prevention or diminution of structural injury and alterations in renal function; the reduction of proteinuria by moderating inflammation through increased mRNA expression of Nrf2, NADPH quinone oxidoreductase 1 (NQO-1), HO-1, and SOD; and decreasing OS. Moreover, the results of many preclinical models of kidney disease suggest that SFN could act on several pathways in kidney damage, particularly in ameliorating inflammation and OS, and thus could represent a strategy of choice to improve the prognosis of CKD patients by preventing the progression of CKD [113,114,115].

In the same way, BS supplements have long been commercialized for the promising health benefits of SFN, which induces the NrF2 pathway and the downstream chemoprotective genes, including Phase 2 enzymes. Most commercially available BS supplements contain BS packaged as glucoraphanin (GR), which is hydrolyzed to SFN by the intestinal microbiota, which synchronously augments the serum activities of Phase 2 enzymes such as NQO1 and glutathione S-transferase (GST), and also Nrf2 signaling, in several human tissues. The researchers suggested that low doses (30 mg per day) of GF have favorable chemoprotective actions in humans [99]. Likewise, the antioxidant effects are attributed to SF, as shown in a randomized placebo-controlled double-blind trial in male patients diagnosed with fatty liver. This research suggested that dietary supplementation with BS extract including the SF precursor GR is likely to be successful in enhancing liver function through reduction of the OS pathway [100]. In the same manner, a clinical study was conducted to evaluate the anti-inflammatory effects of BS powder (BSP) with a high sulforaphane content, in which the researchers analyzed the inflammatory biomarkers in patients diagnosed with T2DM, who were randomly assigned to three treatment groups for 4 weeks. The groups received either 10 g/d BSP (*n* = 27), 5 g/d BSP (*n* = 29), or a placebo (*n* = 25). The results showed that serum high-sensitive C reactive protein (hs-CRP) and interleukin-6 (IL-6) were lower in Group A (10 g/d dose) compared with the control group post-intervention. This indicated that sulforaphane-rich BSP had positive effects on inflammatory biomarkers in patients with T2DM [28]. On the other hand, a clinical study that assessed the actions of prolonged supplementation with BS on inflammatory parameters (TNF-α, IL-6, IL-1β, and CRP) in 40 healthy overweight subjects. The treatment phase involved BS administration at a dose of 30 g per day for 10 weeks and the follow-up phase was 10 weeks of the habitual diet and free ingestion of BS. The results showed that IL-6 and CRP values reduced considerably and throughout the control phase and that the inflammatory marker concentrations were kept low [116]. Additionally, the researchers explored the anti-inflammatory effects of SF in a randomized controlled study in healthy young people and demonstrated the effects of cruciferous vegetables with a high SF content on specific serum inflammatory parameters, particularly the decrease in IL-6, CRP, and soluble TNF receptor (sTNFRI), being careful to mention that these concentrations were higher among persons with *GSTM1*-null genotypes [117].

## 4. Discussion

Renal disease usually develops over time, so it is not often diagnosed until significantly later, when renal function is severely impaired. Pathophysiologically, CKD is a consequence of numerous pathological lesions that abolish some of the nephrons; subsequently, the nephrons overcompensate with hyperfiltration. Over time, glomerular hypertension, albuminuria, and loss of renal activity are established. The increased glomerular capillary pressure triggers damage to the glomerular capillary endothelium, impairment of the podocytes lining the capillaries, and increased permeability of the macromolecules. [118,119]. Additionally, increased pro-inflammatory moderators that induce fibrotic cell proliferation are involved. Furthermore, the increase in ECM molecules gives rise to the development of scarring and renal damage [120]. Currently, there are therapeutic approaches to CKD, with all alternatives targeted at dismissing or preventing deterioration of the disorder, including medication, conservative care, dialysis, and transplantation [121].

Research has recommended that diets rich in vegetables and fruits help to control body weight and defend against chronic disorders, including metabolic and cardiovascular disease, cancer, and CKD [20,122,123]. Only a limited number of trials have analyzed bioactive compound supplementation in humans. Further research must be undertaken to establish the best dosage and method of administration of bioactive compounds [114,115]. Furthermore, future investigations should be directed towards analyzing the particular signaling/cellular mechanisms modulated by bioactive compounds that contribute to the prevention or attenuation of renal damage. Further investigations are also needed to evaluate the pharmacokinetic parameters of bioactive compounds, such as the ideal route of administration, dosage and bioavailability, metabolism, tissue distribution, and clearance, and pharmacodynamic parameters, such as the molecular mechanisms of action of bioactive compounds, to demonstrate their efficacy and safety in the short and long term in clinical studies.

## 5. Conclusions

This review highlights the beneficial function of bioactive medicinal plant compounds on improving renal function. All natural compounds explained in this report appear to have some benefits in improving OS, inflammation, and antioxidant capacity. Several NPCs reduce fibrosis due to hyperglycemia-induced OS, attenuate proteinuria, decrease hematuria and systolic blood pressure, and exert anti-nephrotoxic effects, and thus they are promising therapeutic tools to reduce or prevent the onset and progress of KD pathogenesis. Natural bioactive compounds are considered to exert important actions within the mechanistic pathways and to possess promising clinical properties; however, additional investigations are necessary to obtain a deeper comprehension of their specific pathophysiological functions, and the evaluation of these effects in larger trials is strongly suggested.

## Figures and Tables

**Table 1:** Clinical evidence suggesting the renoprotective effects of bioactive compounds.

Bioactive Compound	Chemical Class and Natural Sources	Study Type	Mechanisms of Renoprotection	Molecular Markers	Refs.
Alliin	Garlic (*Allium sativum* L.), Diallyl thiosulfinate	Clinical trial	Anti-inflammation	↓ IL-6, ↓ CRP, and ↓ ESR	[4]
Astaxanthin (3,3′-dihydroxy-β,β’-carotene-4,4′-dione)	Xanthophyll carotenoid; algae, shrimp, lobster, crab, salmon, and other organisms	Clinical trial	Hypolipidemic effects Hypoglycemic effects	↑ Serum adiponectin ↓ Visceral body fat mass. ↓ TG, ↓ VLDL, ↓ cholesterol, ↓ Systolic blood pressure ↑ BMR	[5]
		Clinical trial	Antioxidation Anti-inflammation	↓ MDA and ↓ IL-6 Downregulation of miR-146a	[6]
Baicalin	Flavonoid; roots of *Scutellaria baicalensis* Georgi	Clinical studies	Attenuate AKI	Decreased BUN and Cr levels	[7]
		Clinical studies	Antioxidation Anti-inflammation	↑ GSH, ↑ SOD ↑ aldose reductase (AR) activity. ↓ NF-κB and VEGF	[8]
Betalains/betacyanins	Red beetroot (betalain), *Opuntia stricta* (betacyanin)	Clinical trial	Anti-inflammation Antioxidation Anti-atherosclerosis	↓ hs-CRP ↓ Hcy levels, ↓ SBP, and ↓ FBG ↓ TC, ↓ TG, ↓ non-HDL-c, and ↓ LDL	[9]
Beetroot juice	*Beta vulgaris* (nitrate (NO_3_) is reduced to nitrite (NO_2_) in the oral cavity and is converted to nitric oxide (NO) in the circulation	Pilot study	Decreased renal resistive index Lowered peripheral blood pressure	↓ cDBP ↓ RRI	[10]
Berberine	Isoquinoline alkaloid; *Coptidis rhizoma* and *Cortex phellodendri*	Clinical trial	Improved diabetic kidney disease	↓ UACR and ↓ serum Cys C	[11]
Cordycepin	*Cordyceps militaris*	Clinical studies	Increased kidney function Improved redox properties Lipid-improving	↓ Urinal protein, ↓ BUN, and ↓ creatinine ↓ Cys C, ↓TG, ↓TC, ↓ LDL-c, and ↑ HDL-C ↓ MPO and MDA ↑ NO and SOD	[12]
Curcumin	Curcuminoid; turmeric (*Curcuma longa*)	Pilot study	Antioxidant Anti-inflammation	↓ NF-kB mRNA expression. ↓ hs-CRP plasma levels	[13]
		Clinical trial	Reduces serum lipids and uric acid concentrations	↓ Serum urea and induced urinary excretion ↓TG, ↓TC, ↓ LDL-c, and ↑ HDL-C	[14]
		Pilot study	Antioxidant	↓ Lipid peroxidation ↑ Antioxidant capacity	[15]
		Clinical trial	Anti-inflammation Attenuated proteinuria Decreased hematuria and systolic blood pressure	↓ TGF-β, ↓ IL-8 ↓ Creatinine	[16]
		Clinical trial	Slowed the progression of CKD	↓ Proteinuria, hematuria, and systolic blood pressure	[17]
		Clinical trial	Decreased uremic pruritus in ESRD patients Anti-inflammation	↓ hs-CRP	[18]
Curcumin/quercetin	Bioflavonoids	Clinical trial	Improved early outcomes in cadaveric renal transplantation	↓ Serum Cr Induction of HO-1 DGF lowered	[19]
Epigallocatechin-3-gallate (EGCG)	Green tea (*Camellia sinensis*), natural polyphenolic component of green tea leaves	Clinical trial	Antiproteinuric effect Anti-apoptotic effect	↓ Albuminuria ↓ Caspase 3	[20]
Epigallocatechin-3-gallate (EGCG)/Amla extract (AE) 1:1	Amla extract (*Emblica officinalis*), the Indian gooseberry	Clinical study	Improved diabetic markers in uremic patients Antioxidant effect Anti-atherosclerosis	↓ AGEs formation ↓ NOx ↓ plasma FRAP HDL and LDL/HDL ratio improved	[21]
Glucoraphanin (GR)	Hydrolyzed to SFN by the intestinal microbiota	Clinical trial	Anti-inflammation Anti-OS	↑ NQO1 and GST	[22]
Pomegranate extract	Pomegranate (*Punica granatum*); polyphenols, alkaloids, and anthocyanins	Pilot study	Antioxidant effect Prevention of nephrolithiasis	Scavenging free radicals ↑ levels of PON1 ↓ SSCaOx	[23]
*Resveratrol* (RSV; trans-3,5,4-trihydroxystilbene)	Phytoalexin; red grapes (*Vitis vinifera* L.), peanuts (*Arachis* spp.), and berries (*Vaccinium* spp.)	Clinical trial	Reduction of urine albumin/creatinine ratio Antioxidant effect	↓ FPG, ↓ HbA1c, ↓ insulin levels and ↓ HOMA-IR. ↑ SOD, GSH-Px, CAT and NO	[24]
		Clinical study	Reduction of insulin resistance and oxidative stress	↑ pAkt:Akt ratio in platelets. ↓ Urinary ortho-tyrosine excretion ↑ Insulin sensitivity	[25]
		Clinical study	↓UF volume and rate Ameliorating angiogenesis	↓ PDE VEGF, Flk-1 and Ang-2 ↑ PDE Tie-2 and Tsp-1	[26]
		Clinical study	Improvement in endothelial function	↑ Brachial flow-mediated dilatation (FMD); ↓LDL-c	[27]
Sulforaphane (1-isothiocyanate-4-methylsulphinylbutane)	Isothiocyanate (organosulfur compound); cruciferous vegetables such as broccoli, brussels sprouts, and cabbages	Clinical trial	Anti-inflammation	↓ hs-CRP and ↓ IL-6	[28]

Abbreviations; IL-6, interleukin 6; CRP, C-reactive protein; ESR, erythrocyte sedimentation rate; TG, triglyceride; VLDL, very low-density lipoprotein; BMR, basal metabolic rate; MDA, malondialdehyde; SOD, superoxide dismutase; GSH-px, glutathione peroxidase; AR, aldose reductase; VEGF, vascular endothelial growth factor; NF-κB, nuclear factor κ-light-chain-enhancer of activated B cells; TGF-β1, transforming growth factor-β1; hs-CRP, highly sensitive C reactive protein; Hcy, homocysteine; SBP, systolic blood pressure; TC, total cholesterol; TG, triglycerides; FBG, fasting blood glucose; LDL-c, low-density lipoprotein cholesterol; non-HDL-c, non-high-density lipoprotein cholesterol; cDBP, central diastolic blood pressure; RRI, renal resistive index; Cys C, serum cystatin C; UACR, urine albumin/creatine ratio; MDA, malondialdehyde; MPO, myeloperoxidase; NO, nitric oxide; IL-8, interleukin 8; HO-1, heme oxygenase-1; DGF, delayed graft function; AGEs, advanced glycation end products; NOx, nitrogen oxides; FRAP, ferric reducing/antioxidant power; GST, glutathione S-transferase; NQO1, NAD(P)H: quinone oxidoreductase 1; PON1, serum paraoxonase1 arylesterase activity; SSCaOx, supersaturation of calcium oxalate; FPG, fasting plasma glucose; HbA1c, glycosylated hemoglobin; HOMA-IR, homoeostasis model assessment of insulin resistance; CAT, catalase; pAkt, phosphorylated protein kinase B; Akt, protein kinase B; PDE, peritoneal dialysate effluent; Tie-2, receptor de angiopoietin; Tsp-1, trombospondina-1; FMD, brachial flow-mediated dilatation; Flk-1, fetal liver kinase-1; Ang-2, angiopoietin-2.

## Data Availability

Not applicable.

## References

[1] Avila-Carrasco L., Majano P., Sánchez-Toméro J.A., Selgas R., López-Cabrera M., Aguilera A., González Mateo G. (2019). Natural Plants Compounds as Modulators of Epitelial-to-Mesenchymal Transition. Front. Pharmacol..

[2] Lv J.C., Zhang L.X. (2019). Prevalence and Disease Burden of Chronic Kidney Disease. Adv. Exp. Med. Biol..

[3] Chen T.K., Knicely D.H., Grams M.E. (2019). Chronic Kidney Disease Diagnosis and Management: A Review. JAMA.

[4] Zare E., Alirezaei A., Bakhtiyari M., Mansouri A. (2019). Evaluating the effect of garlic extract on serum inflammatory markers of peritoneal dialysis patients: A randomized double-blind clinical trial study. BMC Nephrol..

[5] Mashhadi N.S., Zakerkish M., Mohammadiasl J., Zarei M., Mohammadshahi M., Haghighizadeh M.H. (2018). Astaxanthin improves glucose metabolism and reduces blood pressure in patients with type 2 diabetes mellitus. Asia Pac. J. Clin. Nutr..

[6] Shokri-Mashhadi N., Tahmasebi M., Mohammadi-Asl J., Zakerkish M., Mohammadshahi M. (2021). The antioxidant and anti-inflammatory effects of astaxanthin supplementation on the expression of miR-146a and miR-126 in patients with type 2 diabetes mellitus: A randomised, double-blind, placebo-controlled clinical trial. Int. J. Clin. Pract..

[7] Dong S., Sun L. (2013). Traditional Chinese drug baicalin and insulin therapy on pancreatic beta-cell function in newly diagnosed type 2 diabetes. Chin. Med..

[8] Yang M., Kan L., Wu L., Zhu Y., Wang Q. (2019). Effect of baicalin on renal function in patients with diabetic nephropathy and its therapeutic mechanism. Exp. Ther. Med..

[9] Rahimi P., Mesbah-Namin S.A., Ostadrahimi A., Abedimanesh S., Separham A., Asghari jafarabadi M. (2019). Effects of Betalains on Atherogenic Risk Factors in Patients with Atherosclerotic Cardiovascular Disease. Food Funct..

[10] Kemmner S., Lorenz G., Wobst J., Kessler T., Wen M., Günthner R., Stock K., Heemann U., Burkhardt K., Baumann M. (2017). Dietary nitrate load lowers blood pressure and renal resistive index in patients with chronic kidney disease: A pilot study. Nitric Oxide.

[11] Li Z.Y., Liu B., Zhuang X.J., Shen Y.D., Tian H.R., Ji Y., Li L.X., Liu F. (2018). Effects of berberine on the serum cystatin C levels and urine albumin/creatine ratio in patients with type 2 diabetes mellitus. Zhonghua Yi Xue Za Zhi Chin..

[12] Sun T., Dong W., Jiang G., Yang J., Liu J., Zhao L., Ma P. (2019). Cordyceps militaris mejora la enfermedad renal crónica al afectar la vía de señalización redox TLR4/NF-κ B. Oxid Med. Cell Longev..

[13] Alvarenga L., Salarolli R., Cardozo L.F.M.F., Santos R.S., de Brito J.S., Kemp J.A., Reis D., de Paiva B.R., Stenvinkel P., Lindholm B. (2020). Impact of curcumin supplementation on expression of inflammatory transcription factors in hemodialysis patients: A pilot randomized, double-blind, controlled study. Clin. Nutr..

[14] Panahi Y., Kianpour P., Mohtashami R., Jafari R., Simental-Mendía L.E., Sahebkar A. (2016). curcumin lowers serum lipids and uric acid in subjects with nonalcoholic fatty liver disease: A randomized controlled trial. J. Cardiovasc. Pharmacol..

[15] Jiménez-Osorio A.S., García-Niño W.R., González-Reyes S., Álvarez-Mejía A.E., Guerra-León S., Salazar-Segovia J., Falcón I., Montes de Oca-Solano H., Madero M., Pedraza-Chaverri J. (2016). The effect of dietary supplementation with curcumin on redox status and Nrf2 activation in patients with nondiabetic or diabetic proteinuric chronic kidney disease: A Pilot Study. J. Ren. Nutr..

[16] Khajehdehi P., Pakfetrat M., Javidnia K., Azad F., Malekmakan L., Nasab M.H., Dehghanzadeh G. (2011). Oral supplementation of turmeric attenuates proteinuria, transforming growth factor-β and interleukin-8 levels in patients with overt type 2 diabetic nephropathy: A randomized, double-blind and placebo-controlled study. Scand. J. Urol. Nephrol..

[17] Khajehdehi P., Zanjaninejad B., Aflaki E., Nazarinia M., Azad F., Malekmakan L. (2012). Oral supplementation of turmeric decreases proteinuria, hematuria, and systolic blood pressure in patients suffering from relapsing or refractory lupus nephritis: A randomized and placebo-controlled study. J. Ren. Nutr..

[18] Pakfetrat M., Basiri F., Malekmakan L., Roozbeh J. (2014). Effects of turmeric on uremic pruritus in end stage renal disease patients: A double-blind randomized clinical trial. J. Nephrol..

[19] Shoskes D., Lapierre C., Cruz-Correa M., Muruve N., Rosario R., Fromkin B., Braun M., Copley J. (2005). Beneficial effects of the bioflavonoids curcumin and quercetin on early function in cadaveric renal transplantation: A randomized placebo controlled trial. Transplantation.

[20] Borges C.M., Papadimitriou A., Duarte D.A., Lopes de faria J.M., Lopes de faria J.B. (2016). The use of green tea polyphenols for treating residual albuminuria in diabetic nephropathy: A double-blind randomised clinical trial. Sci. Rep..

[21] Chen T.-S., Liou S.-Y., Wu H.-C., Tsai F.-J., Tsai C.-H., Huang C.-Y., Chang Y.-L. (2011). Efficacy of Epigallocatechin-3-Gallate and Amla (*Emblica officinalis*) Extract for the Treatment of Diabetic-Uremic Patients. J. Med. Food.

[22] Ushida Y., Suganuma H., Yanaka A. (2015). Low-dose of the sulforaphane precursor glucoraphanin as a dietary supplement induces chemoprotective enzymes in humans. Food Nutr. Sci..

[23] Tracy C.R., Henning J.R., Newton M.R., Aviram M., Zimmerman M.B. (2014). Oxidative stress and nephrolithiasis: A comparative pilot study evaluating the effect of pomegranate extract on stone risk factors and elevated oxidative stress levels of recurrent stone formers and controls. Urolithiasis.

[24] Sattarinezhad A., Roozbeh J., Shirazi Yeganeh B., Omrani G.R., Shams M. (2019). Resveratrol reduces albuminuria in diabetic nephropathy: A randomized double-blind placebo-controlled clinical trial. Diabetes Metab..

[25] Brasnyó P., Molnár G.A., Mohás M., Markó L., Laczy B., Cseh J., Mikolás E., Szijártó I.A., Mérei A., Halmai R. (2011). Resvera-trol improves insulin sensitivity, reduces oxidative stress and activates the akt pathway in type 2 diabetic patients. Br. J. Nutr..

[26] Lin C.-T., Sun X.-Y., Lin A.-X. (2016). Supplementation with high-dose trans-resveratrol improves ultrafiltration in peritoneal dialysis patients: A prospective, randomized, double-blind study. Ren. Fail..

[27] Marques B.C.A.A., Trindade M., Aquino J.C.F., Cunha A.R., Gismondi R.O., Neves M.F., Oigman W. (2018). Beneficial effects of acute trans-resveratrol supplementation in treated hypertensive patients with endothelial dysfunction. Clin. Exp. Hypertens..

[28] Mirmiran P., Bahadoran Z., Hosseinpanah F., Keyzadc A., Azizid F. (2012). Effects of broccoli sprout with high sulforaphane concentration on in-flammatory markers in type 2 diabetic patients: A randomized double-blind placebo-controlled clinical trial. J. Funct. Foods.

[29] Cockwell P., Fisher L.-A. (2020). The global burden of chronic kidney disease. Lancet.

[30] Webster A.C., Nagler E.V., Morton R.L., Masson P. (2017). Chronic kidney disease. Lancet.

[31] Kovács N., Nagy A., Dombrádi V., Bíró K. (2021). Inequalities in the Global Burden of Chronic Kidney Disease Due to Type 2 Diabetes Mellitus: An Analysis of Trends from 1990 to 2019. Int. J. Environ. Res. Public Health.

[32] Jha V., Garcia-Garcia G., Iseki K., Li Z., Naicker S., Plattner B., Saran R., Wang A.Y., Yang C.W. (2013). Chronic kidney disease: Global dimension and perspectives. Lancet.

[33] Minutolo R., Lapi F., Chiodini P., Simonetti M., Bianchini E., Pecchioli S., Cricelli I., Cricelli C., Piccinocchi G., Conte G. (2014). Risk of ESRD and death in patients with CKD not referred to a nephrologist: A 7-year prospective study. Clin. J. Am. Soc. Nephrol..

[34] De Nicola L., Donfrancesco C., Minutolo R., Lo Noce C., De Curtis A., Palmieri L., Iacoviello L., Conte G., Chiodini P., Sorrentino F. (2011). Epidemiology of chronic kidney disease in Italy: Current state and contribution of the CARHES study. G. Ital. Nefrol..

[35] Kidney Disease: Improving Global Outcomes (KDIGO) CKD Work Group (2013). KDIGO clinical practice guideline for the evaluation and management of chronic kidney disease. Kidney Int Suppl..

[36] da Silveira K.D., Pompermayer Bosco K.S., Diniz L.R., Carmona A.K., Cassali G.D., Bruna-Romero O., de Sousa L.P., Teixeira M.M., Santos R.A., Simões e Silva A.C. (2010). ACE2-angiotensin-(1-7)-Mas axis in renal ischaemia/reperfusion injury in rats. Clin. Sci..

[37] Mezzano S.A., Ruiz-Ortega M., Egido J. (2001). Angiotensin II and renal fibrosis. Hypertension.

[38] Navar L.G. (2014). Intrarenal renin-angiotensin system in regulation of glomerular function. Curr. Opin. Nephrol. Hypertens..

[39] Lv L.-L., Liu B.-C. (2015). Role of non-classical renin-angiotensin system axis in renal fibrosis. Front. Physiol..

[40] Yamagishi S., Matsui T. (2010). Advanced glycation end products, oxidative stress and diabetic nephropathy. Oxid. Med. Cell Longev..

[41] Cellek S. (2004). Point of NO return for nitrergic nerves in diabetes: A new insight into diabetic complications. Curr. Pharm. Des..

[42] Denis U., Lecomte M., Paget C., Ruggiero D., Wiernsperger N., Lagarde M. (2002). Advanced glycation end-products induce apoptosis of bovine retinal pericytes in culture: Involvement of diacylglycerol/ceramide production and oxidative stress induction. Free Radic. Biol. Med..

[43] Ramasamy R., Vannucci S.J., Yan S.S.D., Herold K., Yan S.F., Schmidt A.M. (2005). Advanced glycation end products and RAGE: A common thread in aging, diabetes, neurodegeneration, and inflammation. Glycobiology.

[44] Raghavan C.T., Nagaraj R.H. (2016). AGE-RAGE interaction in the TGFβ2-mediated epithelial to mesenchymal transition of human lens epithelial cells. Glycoconj. J..

[45] Wautier M.P., Chappey O., Corda S., Stern D.M., Schmidt A.M., Wautier J.L. (2001). Activation of NADPH oxidase by AGE links oxidant stress to altered gene expression via RAGE. Am. J. Physiol. Endocrinol. Metab..

[46] Panizo S., Martínez-Arias L., Alonso-Montes C., Cannata P., Martín-Carro B., Fernández-Martín J.L., Naves-Díaz M., Carrillo-López N., Cannata-Andía J.B. (2021). Fibrosis in Chronic Kidney Disease: Pathogenesis and Consequences. Int. J. Mol. Sci..

[47] Weidinger A., Kozlov A.V. (2015). Biological activities of reactive oxygen and nitrogen species: Oxidative stress versus signal transduction. Biomolecules.

[48] Kim Y.M., Cho M. (2014). Activation of NADPH oxidase subunit NCF4 induces ROS-mediated EMT signaling in HeLa cells. Cell. Signal..

[49] Shin J.H., Kim K.M., Jeong J.U., Shin J.M., Kang J.H., Bang K., Kim J.H. (2019). Nrf2-Heme Oxygenase-1 Attenuates High-Glucose-Induced Epithelial-to-Mesenchymal Transition of Renal Tubule Cells by Inhibiting ROS-Mediated PI3K/Akt/GSK-3β Signaling. J. Diabetes Res..

[50] Margetts P.J., Bonniaud P., Liu L., Hoff C.M., Holmes C.J., West-Mays J.A., Kelly M.M. (2005). Transient overexpression of TGF-(beta)1 induces epithelial mesenchymal transition in the rodent peritoneum. J. Am. Soc. Nephrol..

[51] Chapman A. (2011). Epithelial-mesenchymal interactions in pulmonary fibrosis. Ann. Rev. Physiol..

[52] Derynck R., Muthusamy B.P., Saeteurn K.Y. (2014). Signaling pathway cooperation in TGF-beta-induced epithelial–mesenchymal transition. Curr. Opin. Cell Biol..

[53] Lamouille S., Xu J., Derynck R. (2014). Molecular mechanisms of epithelial–mesenchymal transition. Nat. Rev. Mol. Cell Biol..

[54] Ishikawa F., Kaneko E., Sugimoto T., Ishijima T., Wakamatsu M., Yuasa A., Sampei R., Mori K., Nose K., Shibanuma M. (2014). A mitochondrial thioredoxin-sensitive mechanism regulates TGF-beta-mediated gene expression associated with epithelial–mesenchymal transition. Biochem. Biophys. Res. Commun..

[55] Boudreau H.E., Casterline B.W., Rada B., Korzeniowska A., Leto T.L. (2012). Nox4 involvement in TGF-beta and SMAD3-driven induction of the epithelial-to-mesenchymal transition and migration of breast epithelial cells. Free Radic. Biol. Med..

[56] Hiraga R., Kato M., Miyagawa S., Kamata T. (2013). Nox4-derived ROS signaling contributes to TGF-β-induced epithelial-mesenchymal transition in pancreatic cancer cells. Anticancer Res..

[57] Rhyu D.Y., Park J., Sharma B.R., Ha H. (2012). Role of Reactive Oxygen Species in Transforming Growth Factor-Beta1–Induced Extracellular Matrix Accumulation in Renal Tubular Epithelial Cells. Transplant. Proc..

[58] Liu X.X., Zhou H.J., Cai L., Zhang W., Ma J.L., Tao X.J., Yu J.N. (2014). NADPH oxidase-dependent formation of reactive oxygen species contributes to transforming growth factor beta1-induced epithelial–mesenchymal transition in rat peritoneal mesothelial cells, and the role of astragalus intervention. Chin. J. Integr. Med..

[59] Lee J.H., Kim J.H., Kim J.S., Chang J.W., Kim S.B., Park J.S., Lee S.K. (2013). AMP-activated protein kinase inhibits TGF-beta-, angiotensin II-, aldosterone-, high glucose-, and albumin-induced epithelial–mesenchymal transition. Am. J. Physiol. Ren. Physiol..

[60] Lawson L.D., Lawson L.D., Bauer R., Lawson L.D. (1998). Garlic: A Review of Its Medicinal Effects and Indicated Active Compounds. Phytomedicines of Europe.

[61] Borlinghaus J., Albrecht F., Gruhlke M.C.H., Nwachukwu I.D., Slusarenko A.J. (2014). Allicin: Chemistry and biological properties. Molecules.

[62] Arreola R., Quintero-Fabián S., López-Roa R.I., Flores-Gutiérrez E.O., Reyes-Grajeda J.P., Carrera-Quintanar L., Ortuño-Sahagún D. (2015). Immunomodulation and anti-inflammatory effects of garlic compounds. J. Immunol. Res..

[63] Prasad K., Laxdal V.A., Yu M., Raney B.L. (1995). Antioxidant activity of allicin, an active principle in garlic. Mol. Cell Biochem..

[64] Sharma N. (2019). Efficacy of garlic and onion against virus. Int. J. Res. Pharm. Sci..

[65] Higuera-Ciapara I., Félix-Valenzuela L., Goyocoolea F.M. (2006). Astaxanthin: A review of its chemistry and applications. Crit. Rev. Food Sci. Nutr..

[66] Guerin M., Huntley M.E., Olaizola M. (2003). Haematococcus astaxanthin: Applications for human health and nutrition. Trends Biotechnol..

[67] Andersen L.P., Holck S., Kupcinskas L., Kiudelis G., Jonaitis L., Janciauskas D., Permin H., Wadstrom T. (2007). Gastric inflammatory markers and interleukins in patients with functional dyspepsia treated with astaxanthin. FEMS Immunol. Med. Microbiol..

[68] Iwamoto T., Hosoda K., Hirano R., Kurata H., Matsumoto A., Miki W., Kamiyama M., Itakura H., Yamamoto S., Kondo K. (2000). Inhi-bition of low-density lipoprotein oxidation by astaxanthin. J. Atheroscler. Thromb..

[69] Fassett R.G., Healy H., Driver R., Robertson I.K., Geraghty D.P., Sharman J.E., Coombes J.S. (2008). Astaxanthin vs. placebo on arterial stiffness, oxidative stress and inflammation in renal transplant patients (Xanthin): A randomised controlled trial. BMC Nephrol..

[70] Coombes J.S., Sharman J.E., Fassett R.G. (2015). Astaxanthin has no effect on arterial stiffness, oxidative stress, or inflammation in renal transplant recipients: A randomized controlled trial (the XANTHIN trial). Am. J. Clin. Nutr..

[71] Diao W., Chen W., Cao W., Yuan H., Ji H., Wang T., Chen W., Zhu X., Zhou H., Guo H. (2019). Astaxanthin protects against renal fibrosis through inhibiting myofibroblast activation and promoting CD8+ T cell recruitment. Biochim. Biophys. Acta Gen. Subj..

[72] Li L., Chen Y., Jiao D., Yang S., Li L., Li P. (2020). Protective Effect of Astaxanthin on Ochratoxin A-Induced Kidney Injury to Mice by Regulating Oxidative Stress-Related NRF2/KEAP1 Pathway. Molecules.

[73] Yu F.Y., Huang S.G., Zhang H.Y., Ye H., Chi H.G., Zou Y., Lv R.X., Zheng X.B. (2014). Effects of baicalin in CD4 + CD29 + T cell subsets of ulcerative colitis patients. World J. Gastroenterol..

[74] Li F., He M., Li R. (2011). Influence of baicalin and telbivudine on chronic hepatitis B cirrhosis and early serum indexes of liver fibrosis. Med. J. West China.

[75] Liu C., Li P. (2001). Effects of baicalin on erythrocyte aldose reductase activity and early diabetes nephropathy. Chin. J. Gerontol..

[76] Zhu Y., Fu Y., Lin H. (2016). Baicalin Inhibits Renal Cell Apoptosis and Protects Against Acute Kidney Injury in Pediatric Sepsis. Med. Sci. Monit..

[77] Gandía-Herrero F., Jiménez-Atiénzar M., Cabanes J., García-Carmona F., Escribano J. (2010). Stabilization of the Bioactive Pigment of Opuntia Fruits through Maltodextrin Encapsulation. J. Agric. Food Chem..

[78] Sarnak M.J., Amann K., Bangalore S., Cavalcante J.L., Charytan D.M., Craig J.C., Gill J.S., Hlatky M.A., Jardine A.G., Landmesser U. (2019). Conference Participants. Chronic Kidney Disease and Coronary Artery Disease: JACC State-of-the-Art Review. J. Am. Coll. Cardiol..

[79] Naqvi S.F.H., Husnain M. (2020). Betalains: Potential Drugs with Versatile Phytochemistry. Crit. Rev. Eukaryot. Gene Expr..

[80] Madadi E., Mazloum-Ravasan S., Yu J.S., Ha J.W., Hamishehkar H., Kim K.H. (2020). Therapeutic Application of Betalains: A Review. Plants.

[81] Sahebkar A., Watts G.F. (2017). Mode of action of berberine on lipid metabolism: A new-old phytochemical with clinical applications?. Curr. Opin. Lipidol..

[82] Yin J., Gao Z., Liu D., Liu Z., Ye J. (2008). Berberine improves glucose metabolism through induction of glycolysis. Am. J. Physiol. Endocrinol. Metabol..

[83] Ni W., Ding H., Tang L. (2015). Berberine as a promising anti-diabetic nephropathy drug: An analysis of its effects and mechanisms. Eur. J. Pharmacol..

[84] Lin Y., Sheng M., Ding Y., Zhang N., Song Y., Du H., Lu N., Yu W. (2018). Berberine protects renal tubular cells against hypoxia/reoxygenation injury via the Sirt1/p53 pathway. J. Nat. Med..

[85] Yoon J.Y., Kim J.H., Baek K.S., Kim G.S., Lee S.E., Lee D.Y., Choi J.H., Kim S.Y., Park H.B., Sung G.H. (2015). A direct protein kinase B-targeted anti inflammatory activity of cordycepin from artifi-cially cultured fruit body of Cordyceps militaris. Pharm. Mag..

[86] Lee C.T., Huang K.S., Shaw J.F., Chen J.R., Kuo W.S., Shen G., Grumezescu A.M., Holban A.M., Wang Y.T., Wang J.S. (2020). Trends in the Immunomodulatory Effects of Cordyceps militaris: Total Extracts, Polysaccharides and Cordycepin. Front. Pharmacol..

[87] Cui J.D. (2015). Biotechnological production and applications of Cordyceps militaris, a valued traditional Chinese medicine. Crit. Rev. Biotechnol..

[88] Liu C., Song J., Teng M., Zheng X., Li X., Tian Y., Pan M., Li Y., Lee R.J., Wang D. (2016). Antidiabetic and antinephritic activities of aqueous extract of Cordyceps militaris fruit body in diet-streptozotocin-induced diabetic Sprague Dawley rats. Oxid. Med. Cell Longev..

[89] Gu Y.-Y., Liu X.-S., Huang X.-R., Yu X.-Q., Lan H.-Y. (2020). Diverse Role of TGF-β in Kidney Disease. Front. Cell Dev. Biol..

[90] Aggarwal B.B., Gupta S.C., Sung B. (2013). Curcumin: An orally bioavailable blocker of TNF and other pro-inflammatory biomarkers. Br. J. Pharmacol..

[91] Aydin M., Ozkok E., Ozturk O., Agachan B., Yilmaz H., Yaylim I., Kebabcioglu S., Ispir T. (2007). Relationship between interleukin-8 and the oxidant-antioxidant system in end-stage renal failure patients. Exp. Clin. Transplant..

[92] Zhang D.W., Fu M., Gao S.H., Liu J.L. (2013). Curcumin and diabetes: A systematic review. Evid. Based Complement. Altern. Med..

[93] Vanaie A., Shahidi S., Iraj B., Siadat Z.D., Kabirzade M., Shakiba F., Mohammadi M., Parvizian H. (2019). Curcumin as a major active component of turmeric attenuates proteinuria in patients with overt diabetic nephropathy. J. Res. Med. Sci..

[94] Hidaka H., Ishiko T., Furuhashi T., Kamohara H., Suzuki S., Miyazaki M., Ikeda O., Mita S., Setoguchi T., Ogawa M. (2002). Curcumin inhibits interleukin 8 production and improves interleukin 8 receptor expression on the cell surface: Impact on human pancreatic carcinoma cell growth by autocrine regulation. Cancer.

[95] Wickenberg J., Ingemansson S.L., Hlebowicz J. (2010). Effects of Curcuma longa (turmeric) on postprandial plasma glucose and insulin in healthy subjects. Nutr. J..

[96] Hill-Kapturczak N., Thamilselvan V., Liu F., Nick H.S., Agarwal A. (2001). Mechanism of heme oxygenase-1 gene induction by curcumin in human renal proximal tubule cells. Am. J. Physiol Ren. Physiol..

[97] Balogun E., Hoque M., Gong P., Killeen E., Green C.J., Foresti R., Alam J., Motterlini R. (2003). Curcumin activates the heme oxygenase-1 gene via regulation of Nrf2 and the antioxidant responsive element. Biochem. J..

[98] Weir M.A., Walsh M., Cuerden M.S., Sontrop J.M., Chambers L.C., Garg A.X. (2018). Micro-Particle Curcumin for the Treatment of Chronic Kidney Disease-1: Study Protocol for a Multicenter Clinical Trial. Can. J. Kidney Health Dis..

[99] Kanwar J., Taskeen M., Mohammad I., Huo C., Chan T.H., Dou Q.P. (2012). Recent advances on tea polyphenols. Front. Biosci..

[100] Bao H., Peng A. (2016). The green tea polyphenol (-)-epigallocatechin-3-gallate and its beneficial roles in chronic kidney disease. J. Transl. Int. Med..

[101] Twal M., Kiefer P., Salameh A., Schnabel J., Ossmann S., von Salisch S., Krämer K., Sobiraj A., Kostelka M., Mohr F.W. (2013). Renoprotective effects of epigallocatechin gallate in a small piglet model of extracorporeal circulation. Pharm. Res..

[102] Kanlaya R., Thongboonkerd V. (2019). Protective Effects of Epigallocatechin-3-Gallate from Green Tea in Various Kidney Diseases. Adv. Nutr..

[103] Chen T.S., Liou S.Y., Chang Y.L. (2009). Supplementation of Emblica officinalis (Amla) extract reduces oxidative stress in uremic patients. Am. J. Chin. Med..

[104] Bhandari P.R. (2012). Pomegranate (*Punica granatum* L). Ancient seeds for modern cure? Review of potential therapeutic applica-tions. Int. J. Nutr. Pharmacol. Neurol. Dis..

[105] Tugcu V., Kemahli E., Ozbek E., Arinci Y.V., Uhri M., Erturkuner P., Metin G., Seckin I., Karaca C., Ipekoglu N. (2008). Protective effect of a potent antioxidant, pomegranate juice, in the kidney of rats with nephrolithiasis induced by ethylene glycol. J. Endourol..

[106] Reichert C.O., Levy D., Bydlowski S.P. (2021). Paraoxonase Role in Human Neurodegenerative Diseases. Antioxidants.

[107] Den Hartogh D.J., Tsiani E. (2019). Health Benefits of Resveratrol in Kidney Disease: Evidence from In Vitro and In Vivo Studies. Nutrients.

[108] Saldanha J.F., Leal V.O., Rizzetto F., Grimmer G.H., Ribeiro-Alves M., Daleprane J.B., Carraro-Eduardo J.C., Mafra D. (2016). Effects of Resveratrol Supplementation in Nrf2 and NF-κB Expressions in Nondialyzed Chronic Kidney Disease Patients: A Random-ized, Double-Blind, Placebo-Controlled, Crossover Clinical Trial. J. Ren. Nutr..

[109] Gal R., Deres L., Toth K., Halmosi R., Habon T. (2021). The Effect of Resveratrol on the Cardiovascular System from Molecular Mechanisms to Clinical Results. Int. J. Mol. Sci..

[110] Gowd V., Kang Q., Wang Q., Wang Q., Chen F., Cheng K.W. (2020). Resveratrol: Evidence for Its Nephroprotective Effect in Diabetic Nephropathy. Adv. Nutr..

[111] Fahey J., Talalay P. (1999). Antioxidant function of sulforaphane: A potent inducer of phase 2 detoxyfication enzymes. Food Chem. Toxicol..

[112] Vanduchova A., Anzenbacher P., Anzenbacherova E. (2019). Isothiocyanate from Broccoli, Sulforaphane, and Its Properties. J. Med. Food..

[113] Uddin M.J., Kim E.H., Hannan M.A., Ha H. (2021). Pharmacotherapy against Oxidative Stress in Chronic Kidney Disease: Prom-ising Small Molecule Natural Products Targeting Nrf2-HO-1 Signaling. Antioxidants.

[114] Riedl M.A., Saxon A., Diaz-Sanchez D. (2009). Oral sulforaphane increases Phase II antioxidant enzymes in the human upper airway. Clin. Immunol..

[115] Lopez-Chillon M.T., Carazo-Dıaz C., Prieto-Merino D., Zafrilla P., Moreno D.A., Villaño D. (2019). Effects of long-term consumption of broccoli sprouts on inflammatory markers in overweight subjects. Clin. Nutr..

[116] Navarro S.L., Schwarz Y., Song X., Wang C.Y., Chen C., Trudo S.P., Kristal A.R., Kratz M., Eaton D.L., Lampe J.W. (2014). Cruciferous vegetables have variable effects on biomarkers of systemic inflammation in a randomized controlled trial in healthy young adults. J. Nutr..

[117] Gajjala P.R., Sanati M., Jankowski J. (2015). Cellular and molecular mechanisms of chronic kidney disease with diabetes mellitus and cardiovascular diseases as its comorbidities. Front. Immunol..

[118] Gewin L., Zent R., Pozzi A. (2017). Progression of chronic kidney disease: Too much cellular talk causes damage. Kidney Int..

[119] Zoja C., Abbate M., Remuzzi G. (2015). Progression of renal injury toward interstitial inflammation and glomerular sclerosis is dependent on abnormal protein filtration. Nephrol. Dial. Transplant..

[120] Turner J.M., Bauer C., Abramowitz M.K., Melamed M.L., Hostetter T.H. (2012). Treatment of chronic kidney disease. Kidney Int..

[121] Stefan N., Häring H.-U., Schulze M.B. (2018). Metabolically healthy obesity: The low-hanging fruit in obesity treatment?. Lancet Diabetes Endocrinol..

[122] Kuzma J.N., Schmidt K.A., Kratz M. (2017). Prevention of metabolic diseases: Fruits (including fruit sugars) vs. vegetables. Curr. Opin. Clin. Nutr. Metab. Care.

[123] Vieira A.R., Abar L., Vingeliene S., Chan D.S.M., Aune D., Navarro-Rosenblatt D., Stevens C., Greenwood D., Norat T. (2016). Fruits, vegetables, and lung cancer risk: A systematic review and meta-analysis. Ann. Oncol..

